# Nonlinear association of cardiometabolic index with hyperuricemia: insights from the NHANES 1999-2018 study

**DOI:** 10.3389/fendo.2025.1459946

**Published:** 2025-03-26

**Authors:** Xumei Yang, Yulan Luo, Wei Lai

**Affiliations:** ^1^ Department of Critical Care Medicine, West China Hospital, Sichuan University, Chengdu, China; ^2^ West China School of Nursing, Sichuan University, Chengdu, China

**Keywords:** cardiometabolic index (CMI), hyperuricemia, NHANES (National Health and Nutrition Examination Survey), generalized additive model, nonlinear

## Abstract

**Background:**

Hyperuricemia, a risk factor for gout and cardiovascular diseases, has been linked to various metabolic disorders. This study investigates the association between the cardiometabolic index (CMI) and hyperuricemia.

**Methods:**

Using the National Health and Nutrition Examination Survey 1999-2018 data from 23,212 participants, we employed survey-weighted logistic regression to quantify the CMI-hyperuricemia relationship. Generalized additive models explored potential nonlinear relationships, with two-piecewise logistic regression identifying inflection points. Stratified analyses across demographic and health subgroups assessed relationship consistency.

**Results:**

We found a significant association between higher CMI and increased hyperuricemia and identified a nonlinear relationship, characterized by a faster risk increase at lower CMI levels and slower at higher levels. This pattern remained consistent across all demographic and health subgroups.

**Conclusions:**

Higher CMI significantly predicts hyperuricemia across diverse populations, with a pronounced nonlinear association. This pattern underscores the importance of early intervention, emphasizing the need for personalized risk assessments and targeted strategies.

## 1 Introduction

Hyperuricemia is a prevalent condition worldwide, significantly contributing to the risk of gout, nephrolithiasis, metabolic syndrome, cardiovascular disease, and non-alcoholic fatty liver disease (1). The pathogenesis of hyperuricemia is complex, with obesity being a critical factor. Traditionally, weight loss has been recommended to manage hyperuricemia (2); however, there is a substantial number of asymptomatic adults with normal body weight who still suffer from hyperuricemia (3). This highlights the need for more precise and effective screening tools for hyperuricemia, particularly in populations with normal body mass index (BMI).

The cardiometabolic index (CMI) has emerged as a novel marker integrating waist circumference (WC), triglyceride (TG) levels, high-density lipoprotein cholesterol (HDL-C), and height to evaluate metabolic health more comprehensively than traditional markers such as BMI, which does not differentiate between muscle and adipose tissue (4). Recent studies have indicated that CMI is a superior predictor of metabolic syndrome, diabetes, stroke, and other diseases compared to conventional obesity measures (5–8). Furthermore, CMI has been shown to be associated with cardiovascular disease risk in patients with hypertension and obstructive sleep apnea, underscoring its relevance in cardiovascular health (9). Despite these advancements, the potential of CMI as a predictor of hyperuricemia remains understudied, particularly regarding the relationship between CMI and hyperuricemia across different population subgroups and the possible nonlinear association between CMI and hyperuricemia in nationally representative populations.

Several critical gaps exist in the current literature regarding CMI and hyperuricemia. First, while some studies have examined the linear correlation between CMI and hyperuricemia, these investigations have been limited in scope and have not been conducted in large, nationally representative populations. Second, the potential variation in this relationship across different demographic and health subgroups remains unexplored, limiting our understanding of its clinical applicability across diverse populations. Third, despite emerging evidence suggesting nonlinear relationships between CMI and other metabolic conditions, the potential nonlinear nature of the CMI-hyperuricemia relationship has not been comprehensively examined using advanced statistical methods (10–16).

The investigation of nonlinear relationships between CMI and hyperuricemia is particularly warranted for several reasons. First, biological systems rarely follow strictly linear patterns, and metabolic parameters often demonstrate threshold effects or saturation phenomena (17, 18). This has been demonstrated in studies of other metabolic conditions, where risk relationships show distinct patterns at different exposure levels (19–21). Second, previous research on related metabolic markers has revealed that assuming linearity may oversimplify complex biological relationships and potentially miss critical intervention points. For instance, studies examining adiposity measures and cardiometabolic outcomes have identified threshold effects that significantly impact clinical decision-making (22, 23). Third, understanding the potential nonlinearity of the CMI-hyperuricemia relationship could have important implications for risk assessment and intervention strategies, particularly in identifying high-risk populations and determining optimal intervention timing.

Previous studies have reported linear associations between CMI and hyperuricemia in specific populations (11–13, 15). However, emerging evidence indicates that the relationship between CMI and other metabolic diseases may be nonlinear (19–21). The generalized additive model (GAM) is a statistical tool that can capture these nonlinear associations, providing a more nuanced understanding of the risk dynamics involved (24). This study leverages data from the National Health and Nutrition Examination Survey (NHANES) 1999-2018 to investigate the nonlinear relationship between CMI and hyperuricemia. Using survey-weighted analysis to ensure national representativeness, and by employing both logistic regression and GAMs, the research aims to elucidate how CMI influences hyperuricemia risk and to confirm the persistence of this relationship across different demographic and health subgroups in the US adult population.

## 2 Methods

### 2.1 Study design and participants

This study analyzed data from the NHANES 1999-2018, which included a representative sample of the US population. To account for NHANES’ complex survey design and ensure national representativeness, appropriate sampling weights were applied in all analyses. Participants aged 18 years and older were included in the study, resulting in a total sample size of 23,212 individuals. Several exclusion criteria were applied to ensure data validity and reliability: pregnant women were excluded due to pregnancy-induced physiological changes that could confound the CMI-hyperuricemia relationship; participants with missing data on key variables (WC; height; TG; HDL-C, and uric acid levels) were excluded as complete data for these parameters are essential for calculating CMI and determining hyperuricemia status; and those with extreme outlier values of the CMI (defined as values exceeding 3 standard deviations from the mean) were excluded to minimize the impact of potentially erroneous measurements or recording errors on the analysis.

### 2.2 Demographic characteristics

Demographic characteristics such as age, sex, race/ethnicity, poverty income ratio (PIR), education level, physical activity (measured in weekly metabolic equivalents), smoking status, and alcohol consumption were collected through structured interviews. Additionally, physical examination data included BMI, height, and blood pressure. Laboratory data encompassed fasting glucose, uric acid, fasting TG, fasting total cholesterol, HDL-C, low-density lipoprotein cholesterol (LDL-C), and estimated glomerular filtration rate (eGFR). Medical history covered conditions such as diabetes mellitus, hypertension, and cardiovascular disease (including heart attack, congestive heart failure, coronary heart disease, angina, and stroke).

PIR was calculated by dividing household income by the poverty line, categorizing participants into three income groups: low (below 1.3), medium (1.3 to 3.5), and high (above 3.5). Physical activity was quantified in metabolic equivalents (METs/week) for weekly tasks and classified into low (<600 METs/week), medium (600-1,199 METs/week), and high (≥1,200 METs/week) activity levels. Smoking status was categorized into never-smokers (those who smoked fewer than 100 cigarettes in their lifetime), former-smokers (those who smoked more than 100 cigarettes in their lifetime but had quit at the time of the survey), and current smokers (those who smoked more than 100 cigarettes in their lifetime and continued to smoke at least every few days). Current drinking was classified as heavy (defined as 3 or more drinks per day for women, 4 or more drinks per day for men, or 5 or more binge drinking days per month), moderate (2 or more drinks per day for women, 3 or more drinks per day for men, or 2 or more binge drinking days per month), and mild (any other drinking pattern). eGFR was calculated from serum creatinine levels using the 2009 Chronic Kidney Disease Epidemiology Collaboration (CKD-EPI) formula (25).

### 2.3 Measurement of CMI

The CMI was calculated using the following formula (26):


$$CMI=\frac{WC(cm)}{height(cm)}\times \frac{TG(mmol/L)}{HDL-C(mmol/L)}$$

### 2.4 Measurements and definition of hyperuricemia

Hyperuricemia was defined as serum uric acid levels of 7.0 mg/dL or greater for males and 6.0 mg/dL or greater for females (1). Serum uric acid levels were measured using a colorimetric method as part of the NHANES laboratory tests. This definition is consistent with previous studies and clinical guidelines.

### 2.5 Statistical analysis

All analyses incorporated sampling weights, strata, and cluster variables according to NHANES analytic guidelines to account for the complex survey design and ensure nationally representative estimates. The NHANES sampling weights were specifically designed to account for the complex, multistage probability sampling design, non-response adjustments, and post-stratification to match population distributions. For our analysis spanning multiple survey cycles (1999-2018), we constructed appropriate weights following NHANES analytical guidelines by dividing the 2-year sample weights by the number of combined survey cycles (10 cycles). This approach ensures that the combined estimates remain nationally representative of the US civilian non-institutionalized population while accounting for the unequal probability of selection, non-response bias, and differences in demographic characteristics between the sample and the total US population. For variance estimation, we employed the Taylor linearization method (also known as the delta method), which is the default approach implemented in the survey package in R for complex survey designs. This method was chosen because it efficiently handles the NHANES multistage stratified cluster sampling design and provides robust variance estimates for our survey-weighted analyses without requiring the creation of replicate weights. The Taylor linearization approach appropriately accounts for the design effects in NHANES, resulting in accurate standard errors and confidence intervals that reflect the complex sampling strategy rather than assuming simple random sampling. Descriptive statistics were used to summarize the baseline characteristics of the study participants across CMI tertiles. Continuous variables were presented as weighted means with 95% confidence intervals (CI), and categorical variables were presented as weighted percentages with 95% CI. Differences between groups were assessed using survey-weighted linear regression for continuous variables and survey-weighted Chi-square tests (svytable) for categorical variables. Missing data for covariates were addressed using multiple imputation by chained equations (MICE) to reduce bias and improve the robustness of the analyses. The imputation model was carefully specified to preserve the relationships between variables by including all relevant predictors and outcome variables. We used predictive mean matching (PMM) for continuous variables, logistic regression for binary variables, and polytomous regression for categorical variables, with 5 iterations to ensure convergence. The imputation procedure maintained the complex survey design features by incorporating sampling weights, strata, and cluster variables. The stability of the imputation process was confirmed through convergence diagnostics. To assess the sensitivity of our findings to potential violations of the Missing At Random (MAR) assumption, we conducted pattern-mixture sensitivity analyses for a comprehensive set of key confounding variables, including socioeconomic factors (PIR, education), lifestyle factors (physical activity, smoking, drinking), clinical parameters (eGFR), and metabolic conditions (diabetes status). This approach involved systematically modifying values by applying different offset values (from -0.5 to 0.5 standard deviations) to simulate various Missing Not At Random (MNAR) mechanisms, followed by re-estimation of the primary models to evaluate the robustness of our findings regarding the CMI-hyperuricemia relationship.

The association between CMI and hyperuricemia was analyzed using logistic regression models and GAMs. GAMs were specifically chosen over other nonlinear approaches (such as spline regression or nonlinear mixed-effects models) for several reasons: (1) GAMs offer greater flexibility in modeling complex nonlinear relationships without assuming a specific functional form, which is particularly important given the exploratory nature of our investigation into CMI-hyperuricemia associations; (2) GAMs can automatically determine the optimal degree of smoothing through cross-validation, reducing the risk of overfitting; (3) GAMs readily accommodate our survey-weighted data structure while maintaining interpretability; and (4) GAMs allow for easy visualization of nonlinear relationships through smooth function plots, facilitating the identification of potential threshold effects (24). As a sensitivity analysis to verify the robustness of our findings, we also employed restricted cubic splines (RCS) to model the nonlinear relationship between CMI and hyperuricemia.

To examine potential temporal trends in the relationship between CMI and hyperuricemia across the 20-year study period (1999-2018), we conducted stratified analyses by NHANES survey cycle. The study period was divided into ten 2-year cycles corresponding to the NHANES survey design. We estimated the association between CMI (per one standard deviation increase) and hyperuricemia within each cycle using the same multivariable logistic regression model as in our primary analysis, with full adjustment for potential confounders. Additionally, to formally assess the heterogeneity of this association over time, we tested for interaction between survey cycle and CMI by including a product term in our regression model.

Logistic regression models were used to estimate odds ratios (OR) and 95% CI for hyperuricemia across different CMI levels. Before model construction, the assumptions of logistic regression were carefully verified. The linearity of the logit for continuous predictors was assessed using multivariable fractional polynomial (MFP) analysis, with appropriate transformations applied where necessary. Specifically, eGFR was transformed as (eGFR/100)^-0.5 + log(eGFR/100), BMI as log(BMI/10), CMI as log(CMI+1.6), and age as (age/100)^-1 to ensure linear relationships with the logit of hyperuricemia. Multicollinearity was evaluated using variance inflation factors (VIF) through stepwise selection, with variables showing high collinearity being removed. Model specification was assessed through discrimination (AUC) and internal validation using bootstrap resampling (27).

The final models were adjusted for potential confounders including age, sex, race/ethnicity, PIR, educational level, METs/week, smoking, drinking, BMI, eGFR, diabetes, hypertension, and CVD status. GAMs were employed to explore nonlinear associations and were stratified by demographic and health characteristics to verify the persistence of these relationships. The log likelihood ratio test was used to compare models, and statistical significance was set at P < 0.05. Given the multiple analyses conducted across different models, CMI tertiles, and subgroups, we placed emphasis on the consistency of findings across analytical approaches rather than isolated statistical significance tests. The robustness of associations was evaluated through bootstrap internal validation and consistency of direction and magnitude of effects across analyses, which help mitigate concerns about Type I errors due to multiple testing. All statistical analyses were executed using R (version 4.2.2, http://www.R-project.org) and EmpowerStats (version 4.2, www.empowerstats.com).

## 3 Results

### 3.1 Sample selection and exclusion criteria

**Figure 1:**
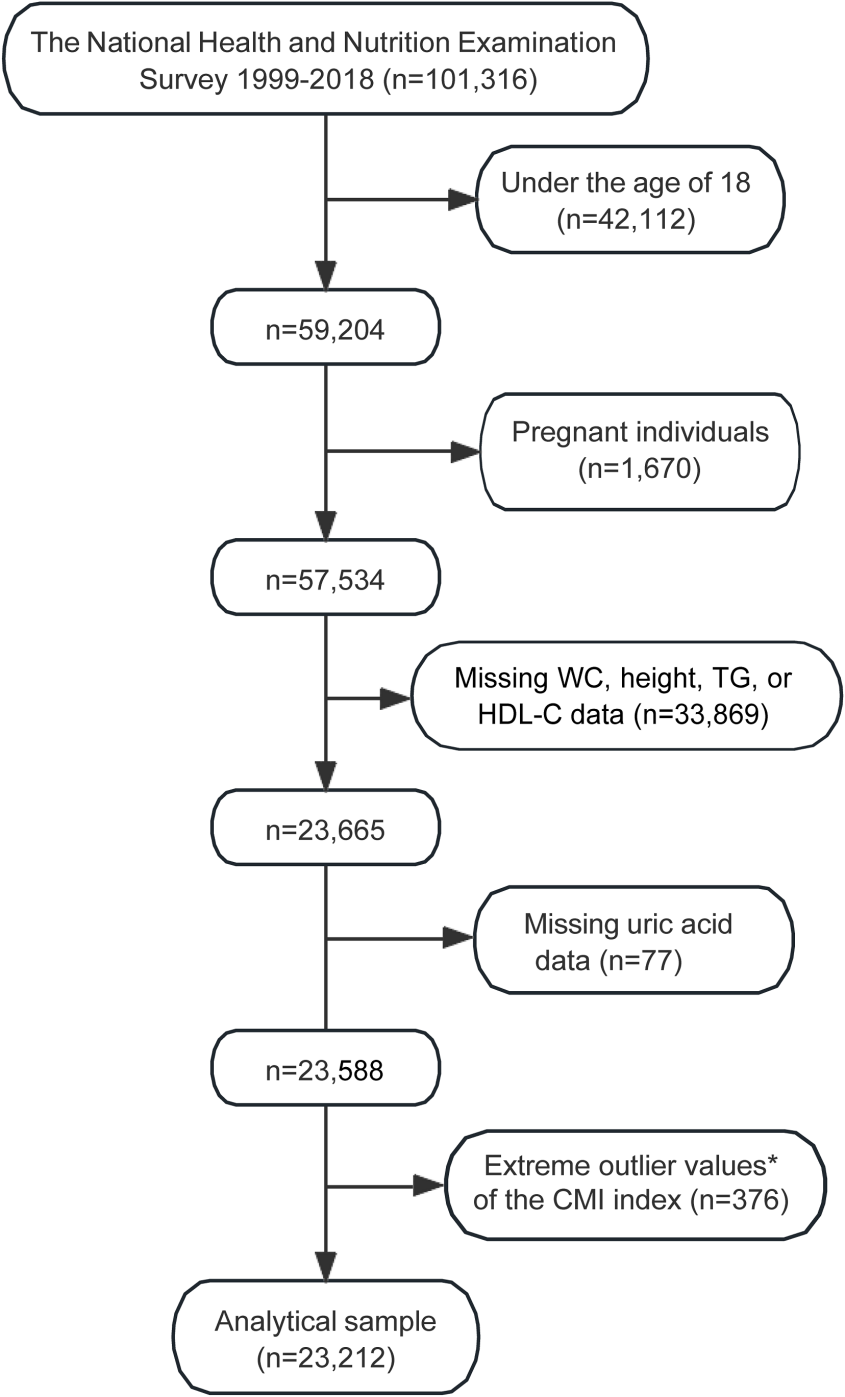
Flowchart illustrating the selection process of the study sample from NHANES 1999–2018. *Extreme outlier values, defined as those over 3 standard deviations from the mean. WC, waist circumference; TG, fasting triglyceride; HDL-C, high-density lipoprotein cholesterol; CMI, cardiometabolic index; NHANES, the National Health and Nutrition Examination Survey.

The selection process for the study sample from the NHANES 1999-2018 is detailed in **Figure 1**. Initially, the dataset included 101,316 participants. Exclusions were made for individuals under the age of 18 (n=42,112) and pregnant individuals (n=1,670), resulting in a reduced sample of 57,534 participants. Further exclusions were necessary due to missing data for key variables such as WC, height, TG, or HDL-C, which accounted for 33,869 participants, reducing the sample to 23,665 individuals.

Additional exclusions were made for those with missing uric acid data (n=77), and extreme outlier values of the CMI (n=376). After applying these exclusion criteria, the final analytical sample consisted of 23,212 participants (**Figure 1**).

### 3.2 Baseline demographic characteristics

The survey-weighted baseline demographic characteristics of the study subjects, categorized into tertiles of the CMI, demonstrate significant differences across various demographic and clinical variables (**Table 1**). Participants in the highest CMI tertile (T3) were older, with a weighted mean age of 49.96 years (95% CI: 49.43, 50.48) compared to 42.36 years (95% CI: 41.71, 43.00) in the lowest tertile (T1) (P < 0.001). The sex distribution showed significant variation; the weighted proportion of males increased from 43.63% (95% CI: 42.20, 45.07) in T1 to 55.91% (95% CI: 54.61, 57.21) in T3, while females decreased from 56.37% (95% CI: 54.93, 57.80) to 44.09% (95% CI: 42.79, 45.39) (P < 0.001). Racial and ethnic composition revealed notable differences, with Non-Hispanic Whites increasing from 66.74% (95% CI: 64.54, 68.86) in T1 to 70.86% (95% CI: 68.38, 73.23) in T3, and Non-Hispanic Blacks decreasing significantly from 14.91% (95% CI: 13.43, 16.52) in T1 to 6.58% (95% CI: 5.77, 7.50) in T3 (P < 0.001). Socioeconomic status, indicated by the PIR, showed a gradient where participants in the low PIR category increased from 19.66% (95% CI: 18.20, 21.21) in T1 to 23.13% (95% CI: 21.79, 24.54) in T3, while those in the high PIR category decreased from 46.23% (95% CI: 44.17, 48.31) to 38.21% (95% CI: 36.26, 40.20). Education levels followed a similar trend, with less than high school education more prevalent in higher CMI tertiles. Physical activity, measured in METs/week, showed significant differences across CMI tertiles. Participants in the highest CMI tertile were more likely to have low physical activity levels (36.54% in T3 *vs*. 29.87% in T1). Moderate physical activity was similarly distributed across tertiles (3.18% in T3 *vs*. 2.78% in T1). Vigorous physical activity was more common in the lowest CMI tertile (67.36% in T1 *vs*. 60.27% in T3). Smoking status varied significantly, with never smokers decreasing from 59.35% (95% CI: 57.48, 61.19) in T1 to 47.18% (95% CI: 45.55, 48.81) in T3. Drinking patterns also differed, with former drinkers being more prevalent in the highest CMI tertile (18.20% *vs*. 10.09% in T1).

**Table 1:** Weighted baseline characteristics of study participants according to CMI tertiles.

Characteristics	CMI tertiles			P-value
	T1 (0.02-0.11) n=7737	T2 (0.11-0.18) n=7737	T3 (0.18-0.57) n=7738	
Age (years)	42.36 (41.71, 43.00)	46.64 (46.06, 47.23)	49.96 (49.43, 50.48)	<0.001
Sex (%)				<0.001
Male	43.63 (42.20, 45.07)	49.16 (47.75, 50.58)	55.91 (54.61, 57.21)	
Female	56.37 (54.93, 57.80)	50.84 (49.42, 52.25)	44.09 (42.79, 45.39)	
Race/ethnicity (%)				<0.001
Non-Hispanic White	66.74 (64.54, 68.86)	68.61 (66.29, 70.85)	70.86 (68.38, 73.23)	
Non-Hispanic Black	14.91 (13.43, 16.52)	10.26 (9.16, 11.48)	6.58 (5.77, 7.50)	
Mexican American	6.04 (5.21, 6.99)	8.67 (7.54, 9.94)	10.12 (8.79, 11.62)	
Others	12.31 (10.96, 13.80)	12.46 (11.21, 13.83)	12.44 (10.99, 14.05)	
PIR (%)				<0.001
Low	19.66 (18.20, 21.21)	21.54 (19.99, 23.18)	23.13 (21.79, 24.54)	
Medium	34.10 (32.51, 35.73)	37.19 (35.65, 38.75)	38.66 (37.03, 40.31)	
High	46.23 (44.17, 48.31)	41.27 (39.17, 43.40)	38.21 (36.26, 40.20)	
Education level (%)				<0.001
Less than high school	13.98 (12.91, 15.12)	18.50 (17.23, 19.85)	21.60 (20.33, 22.93)	
High school	22.66 (21.22, 24.16)	24.70 (23.33, 26.13)	26.63 (25.11, 28.20)	
More than high school	63.36 (61.33, 65.35)	56.79 (54.92, 58.64)	51.77 (49.90, 53.64)	
METs/week (%)				<0.001
Low	29.87 (28.26, 31.52)	34.59 (33.04, 36.17)	36.54 (34.84, 38.28)	
Moderate	2.78 (2.30, 3.36)	2.89 (2.42, 3.45)	3.18 (2.71, 3.73)	
Vigorous	67.36 (65.63, 69.03)	62.52 (60.95, 64.06)	60.27 (58.54, 61.98)	
Smoking (%)				<0.001
Never	59.35 (57.48, 61.19)	53.17 (51.53, 54.80)	47.18 (45.55, 48.81)	
Former	20.81 (19.38, 22.32)	24.26 (22.90, 25.68)	29.92 (28.47, 31.40)	
Now	19.84 (18.46, 21.29)	22.57 (21.03, 24.18)	22.91 (21.69, 24.16)	
Drinking (%)				<0.001
Never	11.25 (10.20, 12.40)	11.60 (10.51, 12.78)	12.04 (11.03, 13.13)	
Former	10.09 (9.19, 11.07)	14.29 (13.23, 15.42)	18.20 (16.84, 19.64)	
Mild	37.20 (35.44, 38.98)	34.82 (33.12, 36.56)	35.46 (33.82, 37.13)	
Moderate	20.90 (19.65, 22.20)	16.55 (15.44, 17.73)	13.39 (12.35, 14.50)	
Heavy	20.56 (19.35, 21.83)	22.74 (21.35, 24.20)	20.91 (19.49, 22.41)	
BMI (kg/m^2^)	24.94 (24.78, 25.10)	28.51 (28.32, 28.71)	32.18 (31.94, 32.43)	<0.001
Height (cm)	168.96 (168.68, 169.25)	168.88 (168.60, 169.15)	169.40 (169.09, 169.71)	0.033
SBP (mmHg)	117.16 (116.66, 117.66)	121.24 (120.71, 121.77)	125.22 (124.75, 125.70)	<0.001
DBP (mmHg)	68.68 (68.27, 69.09)	70.54 (70.14, 70.94)	71.98 (71.57, 72.39)	<0.001
Fasting glucose (mg/dL)	96.96 (96.44, 97.48)	102.70 (101.97, 103.43)	113.71 (112.59, 114.82)	<0.001
Uric acid (mg/dL)	4.94 (4.91, 4.98)	5.45 (5.41, 5.49)	6.01 (5.96, 6.05)	<0.001
Fasting triglyceride (mg/dL)	65.18 (64.49, 65.87)	108.94 (108.01, 109.87)	196.13 (193.27, 198.98)	<0.001
Fasting total cholesterol (mg/dL)	191.26 (190.01, 192.51)	194.81 (193.47, 196.14)	195.51 (194.08, 196.94)	<0.001
HDL-C (mg/dL)	63.90 (63.33, 64.47)	53.05 (52.62, 53.49)	44.02 (43.69, 44.35)	<0.001
LDL-C (mg/dL)	114.34 (113.20, 115.48)	119.96 (118.80, 121.12)	112.26 (111.20, 113.31)	<0.001
eGFR (mL/min/1.73 m^2^)	99.60 (98.81, 100.38)	95.34 (94.60, 96.07)	91.60 (90.81, 92.39)	<0.001
Diabetes (%)				<0.001
No	95.32 (94.75, 95.84)	88.79 (87.86, 89.66)	75.13 (73.83, 76.40)	
Yes	4.68 (4.16, 5.25)	11.21 (10.34, 12.14)	24.87 (23.60, 26.17)	
Hypertension (%)				<0.001
No	77.74 (76.31, 79.10)	64.45 (62.85, 66.02)	50.27 (48.66, 51.88)	
Yes	22.26 (20.90, 23.69)	35.55 (33.98, 37.15)	49.73 (48.12, 51.34)	
CVD (%)				<0.001
No	95.97 (95.36, 96.50)	92.15 (91.34, 92.88)	86.60 (85.60, 87.54)	
Yes	4.03 (3.50, 4.64)	7.85 (7.12, 8.66)	13.40 (12.46, 14.40)	
CMI	0.08 (0.08, 0.08)	0.14 (0.14, 0.14)	0.28 (0.27, 0.28)	<0.001

For continuous variables: survey-weighted mean (95% CI), P-value was by survey-weighted linear regression. For categorical variables: survey-weighted percentage (95% CI), P-value was by survey-weighted Chi-square test (svytable).

CMI, cardiometabolic index; PIR, poverty income ratio; MET, metabolic equivalent of task; BMI, body mass index; SBP, systolic blood pressure; DBP, diastolic blood pressure; HDL-C, high-density lipoprotein cholesterol; LDL-C, low-density lipoprotein cholesterol; eGFR, estimated glomerular filtration rate; CVD, cardiovascular disease.

Laboratory characteristics showed significant variations among the CMI tertiles. Weighted mean BMI increased substantially from 24.94 kg/m² (95% CI: 24.78, 25.10) in T1 to 32.18 kg/m² (95% CI: 31.94, 32.43) in T3 (P < 0.001). Systolic and diastolic blood pressures rose from 117.16 mmHg (95% CI: 116.66, 117.66) and 68.68 mmHg (95% CI: 68.27, 69.09) in T1 to 125.22 mmHg (95% CI: 124.75, 125.70) and 71.98 mmHg (95% CI: 71.57, 72.39) in T3, respectively (P < 0.001). Fasting glucose levels escalated from 96.96 mg/dL (95% CI: 96.44, 97.48) in T1 to 113.71 mg/dL (95% CI: 112.59, 114.82) in T3, and uric acid levels from 4.94 mg/dL (95% CI: 4.91, 4.98) to 6.01 mg/dL (95% CI: 5.96, 6.05) (P < 0.001 for both). Lipid profiles revealed marked deterioration, with fasting TGs increasing dramatically from 65.18 mg/dL (95% CI: 64.49, 65.87) in T1 to 196.13 mg/dL (95% CI: 193.27, 198.98) in T3, while HDL-C levels dropped from 63.90 mg/dL (95% CI: 63.33, 64.47) to 44.02 mg/dL (95% CI: 43.69, 44.35) (P < 0.001). LDL-C exhibited a complex pattern across the CMI tertiles, peaking in the middle tertile (P < 0.001). The eGFR declined from 99.60 mL/min/1.73 m² (95% CI: 98.81, 100.38) in T1 to 91.60 mL/min/1.73 m² (95% CI: 90.81, 92.39) in T3 (P < 0.001). The weighted prevalence of diabetes (24.87% *vs*. 4.68%), hypertension (49.73% *vs*. 22.26%), CVD (13.40% *vs*. 4.03%), and hyperuricemia all increased significantly with higher CMI tertiles (P < 0.001), underscoring the association between higher CMI and adverse health outcomes.

### 3.3 Survey-weighted logistic regression analysis of the relationship between CMI and hyperuricemia

The association of the CMI with hyperuricemia was assessed using three models with survey-weighted analysis, each adjusting for different sets of confounders (**Table 2**). In Model 1, which was unadjusted, the weighted OR per one standard deviation increase in CMI was 1.75 (95% CI: 1.68, 1.82). When adjusted for age and sex in Model 2, the OR slightly decreased to 1.68 (95% CI: 1.61, 1.75). In the fully adjusted Model 3, which controlled for a comprehensive set of variables including age, sex, race/ethnicity, PIR, educational level, physical activity (METs/week), smoking, drinking, BMI, eGFR, diabetes, hypertension, and CVD, the OR further reduced to 1.37 (95% CI: 1.30, 1.44).

**Table 2:** Weighted analysis of the association between CMI and hyperuricemia.

Exposure	Model 1	Model 2	Model 3
CMI (per one SD)	1.75 (1.68, 1.82)	1.68 (1.61, 1.75)	1.37 (1.30, 1.44)
CMI tertiles			
T1 (0.02-0.11)	Reference	Reference	Reference
T2 (0.11-0.18)	2.29 (2.02, 2.59)	2.16 (1.91, 2.45)	1.63 (1.42, 1.86)
T3 (0.18-0.57)	4.87 (4.32, 5.49)	4.36 (3.86, 4.93)	2.47 (2.14, 2.85)
P for trend	<0.001	<0.001	<0.001

Model 1: Non-adjusted.

Model 2: Adjusted for age and sex.

Model 3: Adjusted for age, sex, race/ethnicity, PIR, educational level, METs/week, smoking, drinking, BMI, eGFR, diabetes, hypertension, and CVD.

CMI, cardiometabolic index; SD, standard deviation; PIR, poverty income ratio; MET, metabolic equivalent of task; BMI, body mass index; eGFR, estimated glomerular filtration rate; CVD, cardiovascular disease.

For categorical analysis, CMI values were divided into population-based tertiles (T1: 0.02-0.11, T2: 0.11-0.18, and T3: 0.18-0.57), with approximately equal sample sizes in each group (n≈7737 per tertile). This approach was chosen to provide balanced statistical power across categories while facilitating clinical interpretation of different risk levels. Notably, the upper boundary of the second tertile (0.18) coincides with the inflection point identified in our subsequent nonlinear analysis (Section 3.5), providing statistical validation for this categorization approach. Examining the CMI tertiles, a significant trend was observed across all models. Compared to the reference group (T1), participants in the second tertile (T2) had a 2.29-fold increased risk of hyperuricemia in Model 1 (95% CI: 2.02, 2.59), which decreased to 2.16 (95% CI: 1.91, 2.45) in Model 2 and further to 1.63 (95% CI: 1.42, 1.86) in Model 3. For the highest tertile (T3), the weighted risk was 4.87 times higher (95% CI: 4.32, 5.49) in Model 1, 4.36 times (95% CI: 3.86, 4.93) in Model 2, and 2.47 times (95% CI: 2.14, 2.85) in Model 3 (all with P for trend <0.001).

### 3.4 Model diagnostics and validation

The logistic regression model assumptions were verified through several diagnostic procedures. MFP analysis identified necessary transformations for continuous predictors to ensure linear relationships with the logit of hyperuricemia. After these transformations, all continuous variables showed appropriate linear relationships with the outcome. VIF analysis confirmed the absence of significant multicollinearity in the final model (all VIF values <5), except for the expected correlation between age and eGFR.

To account for the complex survey design, we conducted comprehensive survey-weighted model diagnostics. Survey-weighted Pearson residuals were plotted against fitted values and key predictors (CMI, age, and BMI), which confirmed the absence of systematic patterns (**Supplementary Figure S1**). While a few outliers were observed, the lowest smoothing curves remained close to the zero line, indicating no substantial model misspecification. We also performed survey-weighted influence diagnostics using Cook’s distance measures adapted for complex survey data. A total of 1,704 potentially influential observations (7.3% of the sample) were identified with Cook’s distance values exceeding the threshold of 4/n (**Supplementary Figure S2**). Sensitivity analysis comparing models with and without these influential observations revealed that although the coefficient for CMI increased (from 3.71 to 7.99, a 115.1% increase, **Supplementary Table S1**), the direction and significance of the association remained consistent, with a high correlation between predictions from both models (r = 0.95). This confirms the robustness of our findings regarding the CMI-hyperuricemia relationship despite the presence of influential observations. Additionally, we assessed the survey-weighted goodness-of-fit using the F-adjusted mean residual test and an adapted Hosmer-Lemeshow test (**Supplementary Table S2**). While both tests yielded significant p-values (P < 0.001 and P = 0.002, respectively), suggesting potential areas for model improvement, this is not uncommon in large samples where even small deviations from perfect fit can result in statistical significance. The model’s discrimination ability remained strong despite these limitations.

The final model demonstrated good discrimination with an AUC of 0.7774 and accuracy of 0.808 (95% CI: 0.803-0.813). Internal validation through bootstrap resampling confirmed stable model performance with an accuracy of 0.8075 (SD: 0.0032) and Kappa coefficient of 0.2344 (SD: 0.0106).

### 3.5 Nonlinear relationship between CMI and hyperuricemia

**Figure 2:**
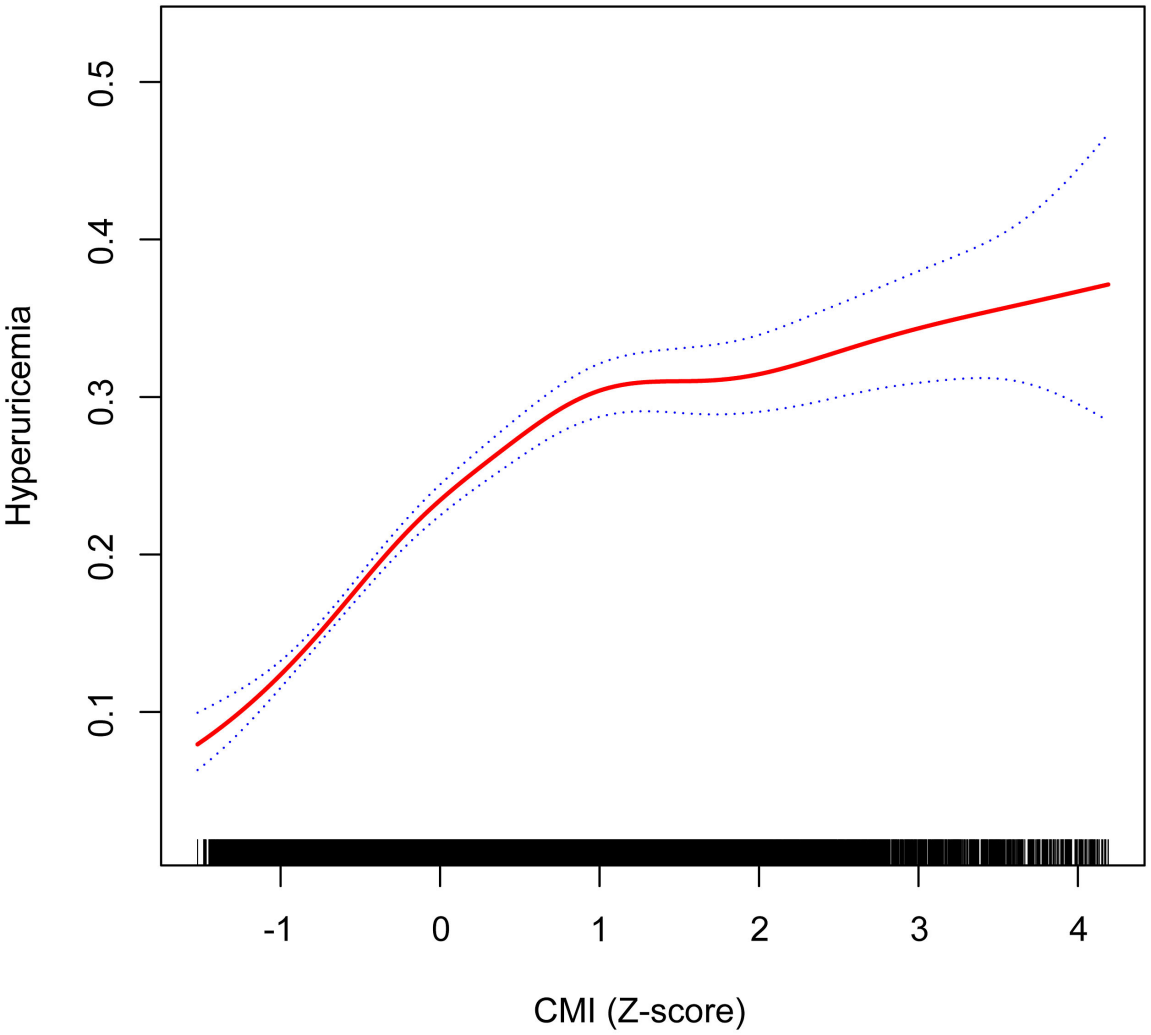
Nonlinear relationship between CMI (Z-score) and hyperuricemia using a GAM, with inflection point at CMI Z-score of 0.11 (original CMI value of 0.18) indicating transition from rapid to gradual risk increase. Age, sex, race/ethnicity, PIR, educational level, METs/week, smoking, drinking, BMI, eGFR, diabetes, hypertension, and CVD were adjusted. The dotted blue lines represent 95% confidence intervals. Below the inflection point, the adjusted OR was 3.57 (95% CI: 2.99, 4.25), while above it, the adjusted OR was 1.30 (95% CI: 1.21, 1.39). CMI, cardiometabolic index; GAM, generalized additive model; PIR, poverty income ratio; MET, metabolic equivalent of task; BMI, body mass index; eGFR, estimated glomerular filtration rate; CVD, cardiovascular disease; OR, odds ratio; CI, confidence interval.

**Figure 2** illustrates the nonlinear relationship between the CMI and the probability of hyperuricemia, using a GAM. The analysis reveals that the risk of hyperuricemia increases more rapidly at lower CMI levels and more slowly at higher CMI levels. The plot shows that initial increases in CMI are associated with a steep rise in hyperuricemia risk, which then tapers off as CMI continues to increase. Two dotted lines represent the 95% CIs, indicating the precision of the estimates and reinforcing the robustness of the observed trend.

The nonlinear relationship between the CMI and hyperuricemia was further explored using a survey-weighted two-piecewise logistic regression model to identify inflection points in the GAM (**Table 3**). The analysis revealed a significant nonlinear association, with an inflection point at a CMI Z-score of 0.11, corresponding to an original CMI value of 0.18. Below this inflection point (CMI Z-score < 0.11), the adjusted OR for hyperuricemia was 3.57 (95% CI: 2.99, 4.25; P < 0.001), indicating a more pronounced increase in risk. Above the inflection point (> 0.18), the adjusted OR was 1.30 (95% CI: 1.21, 1.39; P < 0.001), showing a more modest but still significant increase in risk. The log likelihood ratio test confirmed the significance of this nonlinear relationship (P < 0.001).

**Table 3:** Weighted two-piecewise logistic regression analysis of the association between CMI and hyperuricemia.

CMI (Z-score)	Adjusted OR^*^ (95% CI)	P-value
Model I		
Fitting by the standard linear model	1.37 (1.30, 1.44)	<0.001
Model II		
Inflection point	0.11	
< 0.11	3.57 (2.99, 4.25)	<0.001
> 0.11	1.30 (1.21, 1.39)	<0.001
Log likelihood ratio	/	<0.001

^*^Adjusted for age, sex, race/ethnicity, PIR, educational level, METs/week, smoking, drinking, BMI, eGFR, diabetes, hypertension, and CVD.

CMI, cardiometabolic index; OR, odds ratio; PIR, poverty income ratio; MET, metabolic equivalent of task; BMI, body mass index; eGFR, estimated glomerular filtration rate; CVD, cardiovascular disease.

To verify the robustness of the nonlinear relationship identified by GAM analysis, we conducted a sensitivity analysis using RCS, which confirmed our findings (**Supplementary Figure S3**). The RCS analysis demonstrated a significant nonlinear relationship between CMI and hyperuricemia in both unadjusted (P for overall < 0.01, P for nonlinear < 0.01) and fully adjusted models (P for overall < 0.01, P for nonlinear < 0.01). Similar to our primary GAM analysis, the RCS approach revealed that the risk of hyperuricemia increased rapidly at lower CMI levels and then continued to rise more gradually at higher CMI levels, with an inflection point corresponding to a CMI value of approximately 0.2. This consistency across different statistical methods reinforces the validity of the nonlinear pattern observed in our study.

### 3.6 Stratified analysis of the association between CMI and hyperuricemia

**Figure 3:**
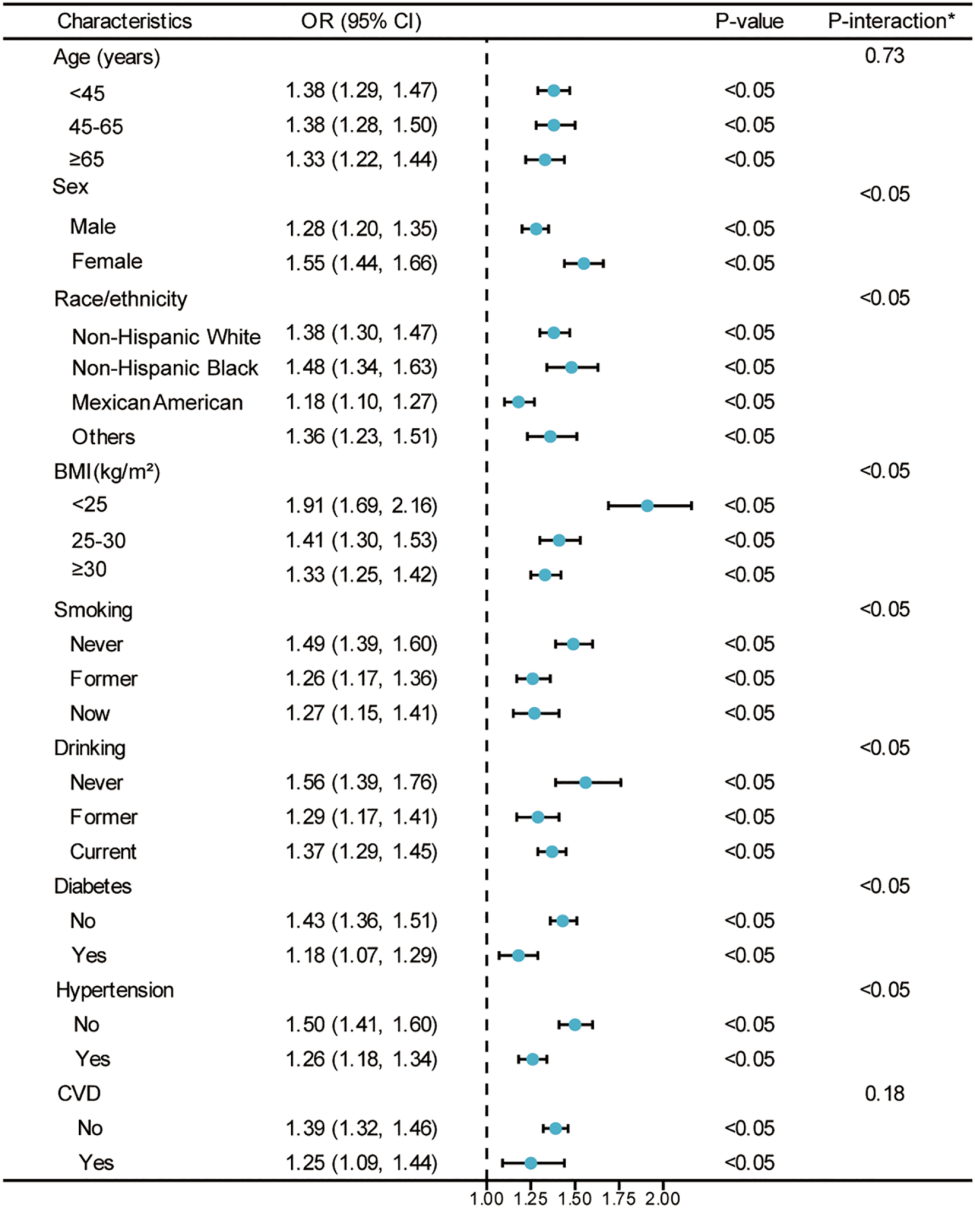
Weighted analysis of the relationship between CMI (Z-score) and hyperuricemia stratified by various demographic and health characteristics. *Each stratification adjusted for all the factors (age, sex, race/ethnicity, PIR, educational level, METs/week, smoking, drinking, BMI, eGFR, diabetes, hypertension, and CVD) except the stratification factor itself. OR, odds ratio; CI, confidence interval; CMI, cardiometabolic index; PIR, poverty income ratio; MET, metabolic equivalent of task; BMI, body mass index; eGFR, estimated glomerular filtration rate; CVD, cardiovascular disease.

In **Figure 3**, the survey-weighted stratified logistic regression models correspond to the relationship verified in **Table 2**. The analysis aims to verify whether there is an interaction between CMI and hyperuricemia within different subgroups. Although most of the interactions among the different subgroups were significant, the predictive significance of CMI for hyperuricemia risk was demonstrated in all subgroups (OR > 1) in the survey-weighted analysis. This analysis illustrates how the weighted OR for hyperuricemia vary with CMI across various demographic and health characteristics, including age, sex, race/ethnicity, BMI categories, smoking status, drinking status, diabetes status, hypertension status, and CVD status. The consistent finding of OR > 1 across all subgroups in the survey-weighted analysis highlights the robust association between higher CMI and increased risk of hyperuricemia, regardless of subgroup differences.

To further examine potential modification effects, we conducted formal tests of interaction (detailed results presented in **Supplementary Table S3**). Significant interactions were found between CMI and sex, race/ethnicity, BMI categories, smoking status, drinking status, diabetes, and hypertension status (all P < 0.05), while no significant interactions were observed for age (P = 0.73) and CVD status (P = 0.18). The CMI-hyperuricemia association was stronger in females compared to males (interaction coefficient: 0.192, SE: 0.041, P < 0.001), but weaker in Mexican Americans compared to Non-Hispanic Whites (interaction coefficient: -0.157, SE: 0.046, P < 0.001). The association decreased with increasing BMI categories and was attenuated in individuals with comorbidities including diabetes (interaction coefficient: -0.197, SE: 0.053, P < 0.001) and hypertension (interaction coefficient: -0.173, SE: 0.041, P < 0.001).

**Figure 4:**
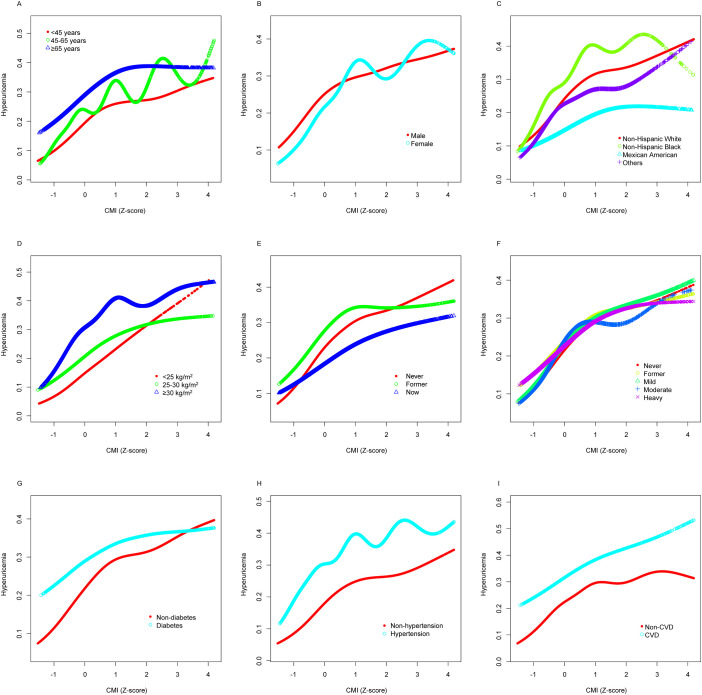
Stratified analyses [by **(A)** age; **(B)** sex; **(C)** race/ethnicity; **(D)** BMI; **(E)** smoking; **(F)** drinking; **(G)** diabetes; **(H)** hypertension; **(I)** CVD] between CMI (Z-score) and hyperuricemia using GAM. Each generalized additive model and smooth curve fitting was adjusted for all factors, including age, sex, race/ethnicity, PIR, educational level, METs/week, smoking, drinking, BMI, eGFR, diabetes, hypertension, and CVD, except for the stratification factor itself. The nonlinear pattern—characterized by faster risk increase at lower CMI levels and slower increase at higher levels—remains consistent across varied subpopulations, confirming the robustness of this relationship. BMI, body mass index; CVD, cardiovascular disease; CMI, cardiometabolic index; GAM, generalized additive model; PIR, poverty income ratio; MET, metabolic equivalent of task; eGFR, estimated glomerular filtration rate.

**Figure 4** employs GAMs to verify whether the nonlinear relationship between CMI and hyperuricemia, presented in **Figure 2**, persists across different subgroups. The hierarchical GAMs illustrate that the probability of hyperuricemia increases with higher CMI scores, but the rate of increase and the shape of the relationship vary among subgroups. Specifically, these models confirm that the nonlinear relationship, where hyperuricemia risk increases faster when CMI is small and slower when CMI is large, holds true within almost each subgroup.

### 3.7 Sensitivity analysis for the MAR assumption

To evaluate the robustness of our findings to potential violations of the MAR assumption, we conducted sensitivity analyses across seven key confounding variables. **Supplementary Table S4** presents the results of these analyses, which simulated various MNAR scenarios by applying different offset values to the imputed data.

The association between CMI and hyperuricemia remained remarkably stable across all MNAR scenarios tested. The original odds ratio for CMI was 5.26 (95% CI: 3.20, 8.65). When simulating different MNAR conditions, the odds ratios exhibited minimal variation, with percent changes ranging from -0.33% to +0.92% across all variables and delta values. eGFR showed the largest impact, with a maximum change of +0.92% (OR: 5.31, 95% CI: 3.23, 8.75), while other variables such as physical activity (MET) showed virtually no impact (percent change: -0.01%). These results confirm that our findings regarding the CMI-hyperuricemia relationship are highly robust to potential violations of the MAR assumption in confounding variables.

### 3.8 Temporal analysis of the CMI-hyperuricemia relationship

**Supplementary Table S5** shows the association between CMI (per one standard deviation increase) and hyperuricemia by NHANES survey cycle from 1999 to 2018. The association remained consistent across most survey cycles, with adjusted odds ratios ranging from 1.11 (95% CI: 0.97-1.27) in 2007-2008 to 1.59 (95% CI: 1.31-1.93) in 2011-2012. Notably, the association was statistically significant (P < 0.05) in all survey cycles except 2007-2008, where it approached but did not reach statistical significance (P = 0.114). The formal test for interaction between survey cycle and CMI revealed no significant temporal variation in this association (P for interaction = 0.067), indicating that the relationship between CMI and hyperuricemia remained largely stable throughout the 20-year study period.

## 4 Discussion

This study analyzed data from NHANES 1999-2018 to investigate the relationship between CMI and hyperuricemia. Using survey-weighted analysis, our major findings reveal a significant nonlinear association, where higher CMI levels correspond to an increased risk of hyperuricemia. This risk increases rapidly at lower CMI levels and more gradually at higher levels. The survey-weighted analysis demonstrated that the association was consistent across all demographic and health subgroups, emphasizing CMI’s robustness as a predictor for hyperuricemia. These results highlight the importance of early intervention and personalized risk assessments to effectively manage hyperuricemia.

The CMI, a relatively new metric related to lipids and obesity, has been linked to various metabolic diseases (5, 6, 26, 28, 29). However, there is a scarcity of studies exploring the relationship between CMI and hyperuricemia. Wang et al. were the first to establish the predictive significance of CMI for hyperuricemia risk in a rural Chinese population (11). Their findings indicated that a one standard deviation increase in CMI corresponded to a 33% increase in the risk of hyperuricemia for both males and females. In comparison, our survey-weighted analysis in the US population showed a more pronounced association, with a 75% increase in hyperuricemia risk per standard deviation increase in CMI in the unadjusted model, and a 37% increase after full adjustment. This difference may be attributed to population characteristics, such as dietary patterns, genetic predispositions, and environmental factors, as well as methodological variations, including the use of survey-weighted analysis in our study. CMI demonstrated similar predictive value for hyperuricemia risk compared to the body adiposity index (BAI) and lipid accumulation product (LAP). Nonetheless, the study’s scope was confined to economically disadvantaged regions, potentially limiting the generalizability of its results. Subsequent research by Liu et al. addressed this limitation by examining a population from the Yangtze River Delta region of China, who regularly underwent physical examinations (15). This broader demographic scope reinforced the earlier findings. Liu et al. determined that among seven nontraditional adiposity indices—BAI, conicity index, a body shape index (ABSI), body roundness index (BRI), visceral adiposity index (VAI), LAP, and CMI—CMI exhibited the strongest association with hyperuricemia and the highest AUC. Similarly, a recent study in hypertensive patients with coronary heart disease identified the metabolic score for visceral fat (METS-VF) index, which integrates visceral obesity and metabolic dysfunction, as the most efficacious predictor of hyperuricemia (30). These findings collectively underscore the robustness of CMI and other obesity metabolism indices as predictors of hyperuricemia, particularly in populations with complex metabolic profiles.

However, both studies were limited to Chinese populations, necessitating further validation for applicability to other ethnic groups. Additionally, our findings extend the literature by demonstrating the robustness of CMI as a predictor of hyperuricemia in a nationally representative US population, highlighting its potential utility across diverse ethnic and demographic groups. In addition to these findings, studies focusing on various subpopulations are noteworthy. Zuo et al. found that CMI correlated more strongly with hyperuricemia than other anthropometric measures in asymptomatic individuals with normal BMI, underscoring the significance of CMI in hyperuricemia management (13). Li et al. later confirmed that CMI had the highest AUC in normotensive hyperuricemic populations (12). Despite these findings, most studies have not examined subgroups beyond those defined by BMI and blood pressure. Furthermore, some researchers have suggested that the association between CMI and other metabolic diseases is nonlinear, rather than simply linear (19–21). This nonlinear relationship is consistent with our findings, as we observed a rapid increase in hyperuricemia risk at lower CMI levels and a more gradual increase at higher levels. Recent studies have further highlighted the importance of considering specific subpopulations and comorbid conditions. For instance, a study in hypertensive patients with coronary heart disease identified the METS-VF index, which integrates visceral obesity and metabolic dysfunction, as the most effective predictor of hyperuricemia, with an AUC of 0.78 (30). This finding underscores the potential value of obesity metabolism indices in populations with complex metabolic profiles, complementing our findings on CMI. Similarly, elevated plasma aldosterone concentrations (PAC) have been shown to exacerbate hyperuricemia by impairing renal uric acid excretion and promoting systemic inflammation, particularly in hypertensive patients (31). These insights suggest that hormonal dysregulation and metabolic dysfunction may play a critical role in the observed associations, particularly in individuals with comorbid conditions such as hypertension and coronary heart disease. Therefore, it is imperative to verify the relationship between CMI and hyperuricemia across different subgroups within the US population and to explore the potential nonlinear relationship between CMI and hyperuricemia.

In our study, based on survey-weighted analysis of a nationally representative sample, the CMI emerged as a significant predictor of hyperuricemia within the US adult population. The association between CMI and hyperuricemia remained statistically significant across various subgroups, including distinctions in age, sex, race/ethnicity, BMI categories, smoking status, drinking status, diabetes status, hypertension status, and CVD status. These findings align with previous studies. In earlier research conducted in China, the United States, and Iran, the TG/HDL-C ratio has been closely linked with the onset and progression of hyperuricemia, serving as a proxy for insulin resistance (32–35). This relationship may be attributed to the fact that elevated TG levels can contribute to the overproduction of uric acid via the free fatty acid metabolic pathway (36). Additionally, low HDL-C levels are independently associated with an increased risk of renal impairment, which can lead to reduced uric acid excretion (37). These mechanisms likely explain the close association between the TG/HDL-C ratio and hyperuricemia. Moreover, the waist-to-height ratio (WHtR), a common measure of obesity, also holds predictive significance for hyperuricemia risk when considered alone (38–41). This is likely because WC is a typical indicator of visceral obesity, and WHtR accounts for height, enhancing its accuracy as a measure of visceral obesity (42). The predictive power of WHtR for hyperuricemia may be due to the fact that visceral fat accumulation can lead to insulin resistance, which affects renal tubules and reduces uric acid excretion (43). In conclusion, CMI, as a novel obesity metric combining the TG/HDL-C ratio and WHtR, may hold greater significance in the management of hyperuricemia.

Our stratified analyses also revealed important variations in the strength of the CMI-hyperuricemia association across population subgroups. The stronger association observed in females compared to males may reflect sex-specific differences in uric acid metabolism, including estrogen’s uricosuric effect and sex-specific fat distribution patterns (44, 45). Women typically have lower baseline uric acid levels, which may make CMI-related metabolic changes more impactful on their relative hyperuricemia risk. The weaker association in Mexican Americans compared to Non-Hispanic Whites which suggests potential ethnic differences in genetic predisposition to hyperuricemia or in dietary patterns affecting uric acid metabolism (46–49). The attenuated association in individuals with higher BMI and those with diabetes or hypertension indicates that once these conditions are established, they may independently influence uric acid levels, partially masking the effect of CMI. In patients with diabetes, for instance, glycosuria may enhance uric acid excretion, potentially counteracting the effects of metabolic dysfunction measured by CMI (50, 51). Similarly, in hypertensive patients, altered renal hemodynamics and medication effects may modify uric acid handling independently of CMI (52–54). These findings highlight the importance of considering individual patient characteristics when using CMI for hyperuricemia risk assessment in clinical practice and suggest that CMI may be particularly valuable as a screening tool in individuals without established metabolic comorbidities.

However, more importantly, we found a nonlinear relationship between CMI and hyperuricemia. The survey-weighted analysis showed that the risk of hyperuricemia increased more rapidly at lower CMI levels and more slowly at higher CMI levels, with an inflection point corresponding to a CMI Z-score of 0.11 and an original CMI value of 0.18. This inflection point coincides with the upper boundary of our second population-based tertile, providing statistical validation for this threshold as a clinically meaningful demarcation. From a clinical perspective, this inflection point distinguishes between distinct metabolic phenotypes. CMI values below 0.18 typically represent individuals with relatively favorable metabolic profiles, including better visceral fat distribution and healthier lipid parameters. Based on the components of CMI calculation, this range generally corresponds to WHtR below 0.5-0.55 (indicating lower visceral adiposity) and triglyceride-to-HDL-C ratios below 3-4 (suggesting better insulin sensitivity). Below this threshold, the adjusted odds ratio was 3.57 (95% CI: 2.99, 4.25), indicating a steep increase in hyperuricemia risk with rising CMI. Conversely, CMI values exceeding 0.18 generally reflect more adverse metabolic parameters, including greater visceral adiposity and more pronounced dyslipidemia, often indicating underlying insulin resistance. Above this threshold, the hyperuricemia risk continues to increase but at a more modest rate (adjusted OR: 1.30, 95% CI: 1.21, 1.39), suggesting potential physiological adaptation mechanisms.

Several biological mechanisms may explain this nonlinear relationship. First, metabolic syndrome progression may play a role (55). At lower CMI levels, individuals may not yet have developed severe metabolic dysfunction, making their physiological systems more sensitive to initial metabolic perturbations. As CMI increases beyond the 0.18 threshold, the body may have already transitioned into a state of metabolic syndrome, potentially activating compensatory mechanisms that moderate the rate of further increase in hyperuricemia risk. These adaptations might include altered renal clearance of uric acid or modified inflammatory pathways. Second, changes in fat distribution and insulin resistance likely contribute to this nonlinear pattern (55–57). Individuals with lower CMI values typically maintain better insulin sensitivity, making their metabolic systems more responsive to small changes. As CMI increases beyond the inflection point, established insulin resistance may render the body less responsive to further metabolic deterioration. Third, hormonal regulation may play a significant role. The initial rise in CMI may trigger sharp increases in leptin levels (stimulating uric acid production) and decreases in adiponectin (impairing insulin sensitivity). Similarly, elevated plasma aldosterone concentrations have been shown to exacerbate hyperuricemia by impairing renal uric acid excretion, particularly in hypertensive patients (31). These hormonal changes may be more pronounced during the transition from metabolically healthy to unhealthy states, and subsequently plateau, thus contributing to the observed nonlinear relationship.

These findings highlight the importance of early intervention and personalized risk assessments to effectively manage hyperuricemia. The identification of a CMI inflection point at 0.18 provides a potential clinical threshold for risk stratification. Patients with CMI values below 0.18 are in a zone of rapidly increasing hyperuricemia risk (OR: 3.57, 95% CI: 2.99, 4.25), suggesting they may benefit from more intensive monitoring and earlier lifestyle interventions to prevent further metabolic deterioration. For individuals with CMI values exceeding 0.18, who already demonstrate a significant hyperuricemia risk, more aggressive management strategies combining lifestyle modifications with consideration of pharmacological interventions may be warranted, particularly if other risk factors are present. In clinical practice, CMI calculation is straightforward, requiring only waist circumference, height, triglyceride, and HDL-C measurements - all routinely collected parameters. This makes CMI an easily implementable tool for hyperuricemia risk assessment in primary care settings. Regular calculation of CMI during routine health examinations could help identify individuals who would benefit from uric acid screening and targeted preventive measures, even before they develop more advanced cardiometabolic complications.

A distinctive aspect of this study is the combination of survey-weighted analysis and the use of GAMs to explore the nonlinear relationship between CMI and hyperuricemia. The survey-weighted approach ensures our findings are representative of the US population, while GAMs allow for more flexible modeling of complex relationships by fitting smooth curves to the data (24). This approach enabled us to detect the rapid increase in hyperuricemia risk at lower CMI levels and the more gradual increase at higher levels, providing a nuanced understanding of how CMI influences hyperuricemia risk. Additionally, our study employed a comprehensive set of demographic and health-related variables to adjust for potential confounders, enhancing the robustness of our findings. The survey-weighted analysis of these factors included age, sex, race/ethnicity, BMI, smoking status, drinking status, physical activity, diabetes, hypertension, and cardiovascular disease status. By using a large, representative sample from NHANES, we ensured that our results are generalizable to the broader US population. The combination of advanced statistical methods and thorough adjustment for confounders underscores the reliability of our findings and highlights the importance of considering nonlinear relationships in epidemiological research.

While our study provides valuable insights into the nonlinear association between CMI and hyperuricemia, there are several limitations to consider. Firstly, the cross-sectional design of NHANES limits our ability to infer causality and prevents us from examining the temporal sequence between CMI changes and hyperuricemia development. This limitation is particularly relevant when interpreting the nonlinear relationship we observed, as we cannot determine whether CMI changes precede or follow changes in uric acid levels. Longitudinal studies with repeated measurements are needed to confirm the temporal relationship between CMI and hyperuricemia. Secondly, our analysis relies on self-reported data for some variables, which may be subject to recall bias and misclassification, particularly for lifestyle factors such as smoking, drinking, and physical activity. The potential misclassification of these important confounders could lead to residual confounding in our analyses. While missing data could potentially introduce bias if the MAR assumption is violated, our comprehensive sensitivity analyses (as presented in Section 3.7) demonstrated that the association between CMI and hyperuricemia remained highly robust under various MNAR scenarios, with minimal changes in the effect estimates. Thirdly, although we adjusted for numerous confounders, residual confounding due to unmeasured variables cannot be ruled out, including but not limited to dietary patterns, medication use (especially uric acid-lowering drugs and diuretics), and genetic factors that might influence both CMI and uric acid levels. Additionally, the single measurement of serum uric acid and anthropometric indices may not fully capture the dynamic nature of these parameters over time. Lastly, despite the use of survey-weighted analysis to improve generalizability, our findings may be limited to the US population, and similar studies in other populations are warranted to validate our results, particularly given the known ethnic differences in body composition and metabolic profiles.

## 5 Conclusion

Based on survey-weighted analysis of NHANES 1999-2018 data, this study highlights the significant role of the CMI as a predictor of hyperuricemia, emphasizing its potential utility in clinical practice for identifying individuals at risk. Our findings revealed a 75% increase in hyperuricemia risk per standard deviation increase in CMI in the unadjusted model, with a nonlinear relationship indicating a more dramatic risk increase at lower CMI levels (OR: 3.57). After adjusting for demographic, clinical, and lifestyle factors, the association remained robust, with a 45% increase in hyperuricemia risk per standard deviation increase in CMI (adjusted OR: 1.45, 95% CI: 1.30–1.62). These results underscore the importance of early intervention and tailored risk management strategies.

Future research should prioritize longitudinal studies to establish causality and validate these findings in diverse populations beyond the US. Additionally, exploring genetic, dietary, and environmental influences will provide a more comprehensive understanding of hyperuricemia’s etiology. For clinical practice, incorporating CMI as a routine screening tool for hyperuricemia risk assessment, particularly in individuals with metabolic syndrome or obesity, could facilitate early identification and timely interventions, such as lifestyle modifications and dietary adjustments. These strategies may help mitigate hyperuricemia progression and reduce the burden of associated metabolic and cardiovascular diseases.

## Data Availability

The raw data supporting the conclusions of this article will be made available by the authors, without undue reservation.
