# Supplementary material for: Nonlinear association of cardiometabolic index with hyperuricemia: insights from the NHANES 1999-2018 study
Source: Front Endocrinol (Lausanne). 2025 Mar 26;16:1459946. doi: 10.3389/fendo.2025.1459946 (PMC11978652; doi:10.3389/fendo.2025.1459946)
Supplement: Supplementary file 1 [file DataSheet1.docx]

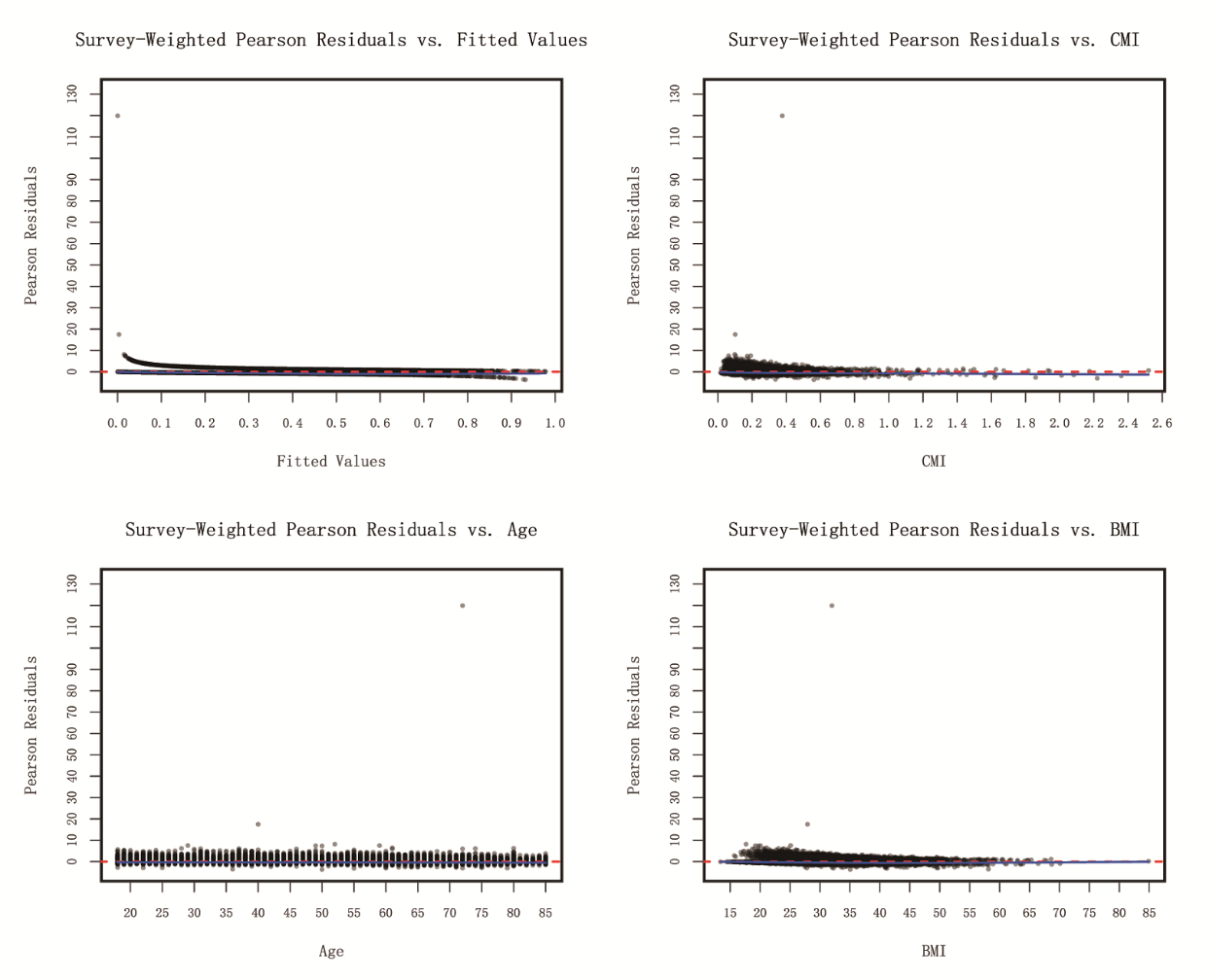


**Figure S1. Survey-weighted Pearson residuals plotted against fitted values and key predictors.**
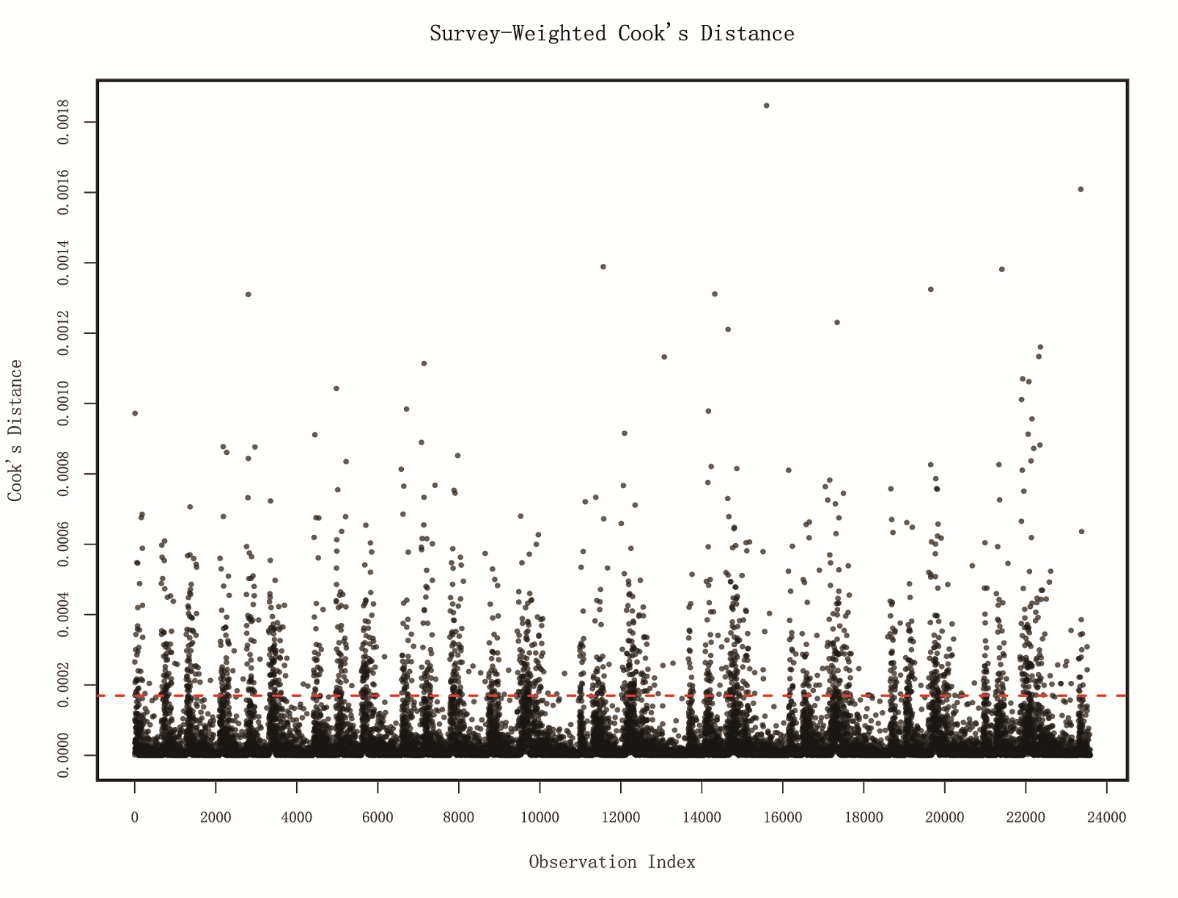


**Figure S2. Survey-weighted Cook's distance plot for identifying influential observations.**


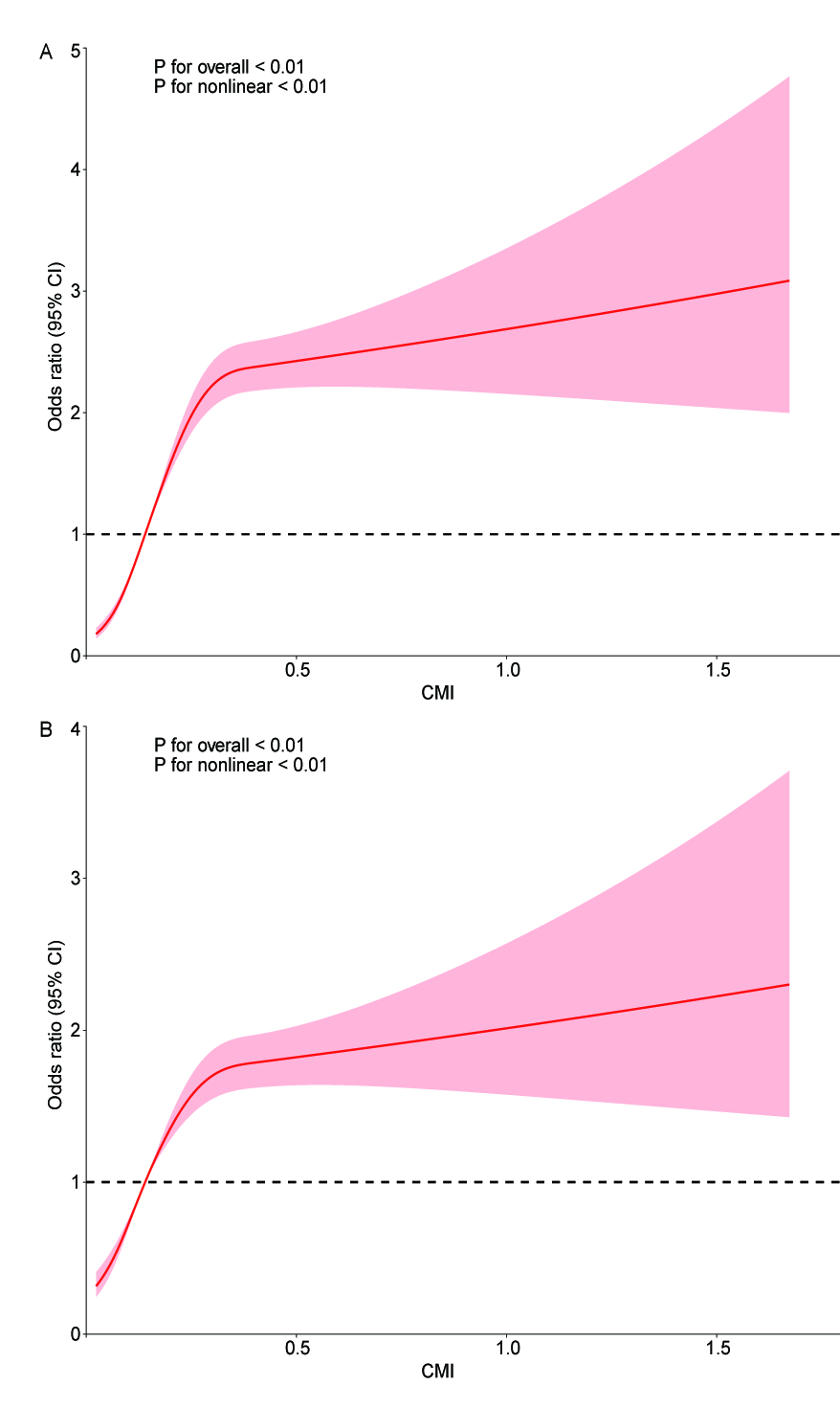


**Figure S3. Nonlinear relationship between CMI and hyperuricemia using restricted cubic splines analysis.**

**Notes**: (A) Unadjusted model showing odds ratios (with 95% CI) for hyperuricemia across CMI values. (B) Fully adjusted model controlling for demographic, lifestyle, and clinical factors. Both models demonstrate significant nonlinear associations (P for nonlinear < 0.01).

**Table S1. Comparison of key model coefficients before and after removing influential observations.**

| **Variable** | **Original Model** | **Filtered Model** | **Absolute Change** | **Percent Change (%)** |
| --- | --- | --- | --- | --- |
| CMI_transformed | 3.71 | 7.99 | 4.28 | 115.11 |

**Table S2. Summary of survey-weighted model fit tests.**

| **Test** | **Statistic** | **P-value** | **Interpretation** |
| --- | --- | --- | --- |
| F-adjusted mean residual test | 32.62 | <0.001 | Potential model fit issues |
| Adapted Hosmer-Lemeshow test | 24.47 | 0.002 | Potential model fit issues |

**Table S3. Survey-weighted logistic regression models with interaction terms for the association between CMI and hyperuricemia**

| **Variables** | **Age interaction** | **Sex interaction** | **Race interaction** | **BMI interaction** | **Smoking interaction** | **Drinking interaction** | **Diabetes interaction** | **Hypertension interaction** | **CVD interaction** |
| --- | --- | --- | --- | --- | --- | --- | --- | --- | --- |
| **Main effects** | | | | | | | | | |
| CMI (per SD) | 0.319 (0.034)*** | 0.244 (0.030)*** | 0.324 (0.032)*** | 0.648 (0.062)*** | 0.402 (0.036)*** | 0.446 (0.060)*** | 0.359 (0.028)*** | 0.406 (0.032)*** | 0.329 (0.027)*** |
| Age (45-65 vs <45) | -0.424 (0.071)*** | - | - | - | - | - | - | - | - |
| Age (≥65 vs <45) | -0.441 (0.096)*** | - | - | - | - | - | - | - | - |
| Sex (Female vs Male) | - | -0.455 (0.047)*** | - | - | - | - | - | - | - |
| Race (Black vs White) | - | - | 0.355 (0.055)*** | - | - | - | - | - | - |
| Race (Mexican vs White) | - | - | -0.116 (0.078) | - | - | - | - | - | - |
| Race (Other vs White) | - | - | 0.168 (0.077)* | - | - | - | - | - | - |
| BMI (25-30 vs <25) | - | - | - | 0.490 (0.066)*** | - | - | - | - | - |
| BMI (≥30 vs <25) | - | - | - | 1.140 (0.061)*** | - | - | - | - | - |
| Smoking (Former vs Never) | - | - | - | - | 0.184 (0.064)** | - | - | - | - |
| Smoking (Current vs Never) | - | - | - | - | -0.098 (0.066) | - | - | - | - |
| Drinking (Former vs Never) | - | - | - | - | - | -0.078 (0.094) | - | - | - |
| Drinking (Current vs Never) | - | - | - | - | - | 0.072 (0.079) | - | - | - |
| Diabetes (Yes vs No) | - | - | - | - | - | - | 0.036 (0.067) | - | - |
| Hypertension (Yes vs No) | - | - | - | - | - | - | - | 0.549 (0.054)*** | - |
| CVD (Yes vs No) | - | - | - | - | - | - | - | - | 0.094 (0.092) |
| **Interaction terms** | | | | | | | | | |
| CMI × Age (45-65) | 0.004 (0.049) | - | - | - | - | - | - | - | - |
| CMI × Age (≥65) | -0.037 (0.054) | - | - | - | - | - | - | - | - |
| CMI × Sex (Female) | - | 0.192 (0.041)*** | - | - | - | - | - | - | - |
| CMI × Race (Black) | - | - | 0.068 (0.055) | - | - | - | - | - | - |
| CMI × Race (Mexican) | - | - | -0.157 (0.046)*** | - | - | - | - | - | - |
| CMI × Race (Other) | - | - | -0.013 (0.060) | - | - | - | - | - | - |
| CMI × BMI (25-30) | - | - | - | -0.302 (0.074)*** | - | - | - | - | - |
| CMI × BMI (≥30) | - | - | - | -0.360 (0.070)*** | - | - | - | - | - |
| CMI × Smoking (Former) | - | - | - | - | -0.171 (0.050)*** | - | - | - | - |
| CMI × Smoking (Current) | - | - | - | - | -0.160 (0.062)* | - | - | - | - |
| CMI × Drinking (Former) | - | - | - | - | - | -0.194 (0.071)** | - | - | - |
| CMI × Drinking (Current) | - | - | - | - | - | -0.134 (0.064)* | - | - | - |
| CMI × Diabetes (Yes) | - | - | - | - | - | - | -0.197 (0.053)*** | - | - |
| CMI × Hypertension (Yes) | - | - | - | - | - | - | - | -0.173 (0.041)*** | - |
| CMI × CVD (Yes) | - | - | - | - | - | - | - | - | -0.102 (0.076) |
| **P-interaction** | 0.73 | <0.05 | <0.05 | <0.05 | <0.05 | <0.05 | <0.05 | <0.05 | 0.18 |

**Notes:** Values are presented as coefficient estimates (standard error).

*P<0.05, **P<0.01, ***P<0.001

All models are adjusted for age, sex, race/ethnicity, poverty income ratio, education level, BMI, smoking status, drinking status, diabetes status, hypertension status, physical activity, eGFR, and cardiovascular disease (except when the variable is included in the interaction term).

**Abbreviations:** SD: standard deviation; CMI: cardiometabolic index.

**Table S4: Sensitivity Analysis for Different Missing Not at Random (MNAR) Assumptions**

| **Variables** | **Delta (SD units)** | **OR (95% CI)** | **% Change from Original** |
| --- | --- | --- | --- |
| Original | 0.00 | 5.26 (3.20, 8.65) | 0.0% |
| PIR | -0.50 | 5.27 (3.22, 8.65) | +0.2% |
| PIR | -0.25 | 5.28 (3.22, 8.65) | +0.2% |
| PIR | 0.00 | 5.28 (3.22, 8.66) | +0.3% |
| PIR | 0.25 | 5.28 (3.22, 8.66) | +0.3% |
| PIR | 0.50 | 5.28 (3.22, 8.67) | +0.4% |
| MET | -0.50 | 5.26 (3.22, 8.61) | 0.0% |
| MET | -0.25 | 5.26 (3.22, 8.61) | 0.0% |
| MET | 0.00 | 5.26 (3.22, 8.61) | 0.0% |
| MET | 0.25 | 5.26 (3.22, 8.61) | 0.0% |
| MET | 0.50 | 5.26 (3.22, 8.61) | 0.0% |
| Education | -0.50 | 5.25 (3.20, 8.63) | -0.2% |
| Education | -0.25 | 5.25 (3.20, 8.63) | -0.2% |
| Education | 0.00 | 5.25 (3.20, 8.63) | -0.2% |
| Education | 0.25 | 5.25 (3.20, 8.63) | -0.2% |
| Education | 0.50 | 5.25 (3.20, 8.63) | -0.2% |
| Smoking | -0.50 | 5.25 (3.20, 8.63) | -0.2% |
| Smoking | -0.25 | 5.25 (3.20, 8.63) | -0.2% |
| Smoking | 0.00 | 5.25 (3.20, 8.63) | -0.2% |
| Smoking | 0.25 | 5.25 (3.20, 8.63) | -0.2% |
| Smoking | 0.50 | 5.25 (3.20, 8.63) | -0.2% |
| Drinking | -0.50 | 5.27 (3.21, 8.64) | +0.1% |
| Drinking | -0.25 | 5.27 (3.21, 8.64) | +0.1% |
| Drinking | 0.00 | 5.27 (3.21, 8.64) | +0.1% |
| Drinking | 0.25 | 5.27 (3.21, 8.64) | +0.1% |
| Drinking | 0.50 | 5.27 (3.21, 8.64) | +0.1% |
| eGFR | -0.50 | 5.31 (3.22, 8.75) | +0.9% |
| eGFR | -0.25 | 5.31 (3.23, 8.75) | +0.9% |
| eGFR | 0.00 | 5.31 (3.23, 8.74) | +0.9% |
| eGFR | 0.25 | 5.31 (3.22, 8.73) | +0.8% |
| eGFR | 0.50 | 5.30 (3.22, 8.72) | +0.6% |
| Diabetes | -0.50 | 5.25 (3.20, 8.60) | -0.3% |
| Diabetes | -0.25 | 5.25 (3.20, 8.60) | -0.3% |
| Diabetes | 0.00 | 5.25 (3.20, 8.60) | -0.3% |
| Diabetes | 0.25 | 5.25 (3.20, 8.60) | -0.3% |
| Diabetes | 0.50 | 5.25 (3.20, 8.60) | -0.3% |

**Note:** Delta represents the offset applied in terms of standard deviation units. Odds ratios represent the association between CMI and hyperuricemia after applying the MNAR assumption to the specified variable.

**Table S5. Association Between Cardiometabolic Index (per one SD increase) and Hyperuricemia by Survey Cycle, NHANES 1999-2018**

| **Survey Cycle** | **Years** | **Adjusted OR* (95% CI)** | **P-value** | **Sample Size** |
| --- | --- | --- | --- | --- |
| Cycle 1 | 1999-2000 | 1.37 (1.04, 1.79) | 0.026 | 2,400 |
| Cycle 2 | 2001-2002 | 1.41 (1.18, 1.67) | <0.001 | 2,215 |
| Cycle 3 | 2003-2004 | 1.38 (1.12, 1.68) | 0.007 | 2,095 |
| Cycle 4 | 2005-2006 | 1.42 (1.13, 1.80) | 0.008 | 2,052 |
| Cycle 5 | 2007-2008 | 1.11 (0.97, 1.27) | 0.114 | 2,476 |
| Cycle 6 | 2009-2010 | 1.33 (1.17, 1.50) | 0.002 | 2,675 |
| Cycle 7 | 2011-2012 | 1.59 (1.31, 1.93) | <0.001 | 2,338 |
| Cycle 8 | 2013-2014 | 1.31 (1.18, 1.46) | <0.001 | 2,478 |
| Cycle 9 | 2015-2016 | 1.51 (1.25, 1.82) | 0.001 | 2,194 |
| Cycle 10 | 2017-2018 | 1.46 (1.22, 1.74) | 0.005 | 2,289 |
| Overall | 1999-2018 | 1.37 (1.32, 1.42) | <0.001 | 23,212 |

**Notes**: *Adjusted for age, sex, race/ethnicity, poverty income ratio, education level, physical activity, smoking status, alcohol consumption, body mass index, estimated glomerular filtration rate, diabetes, hypertension, and cardiovascular disease.

P-value for interaction between survey cycle and CMI = 0.067, indicating no significant temporal variation in the association between CMI and hyperuricemia across survey cycles.

**Abbreviations**: CI, confidence interval; NHANES, National Health and Nutrition Examination Survey; OR, odds ratio; SD, standard deviation.
